# Changes in Accelerometer-measured Physical Activity and Sedentary Behavior From Before to After COVID-19 Outbreak in Workers

**DOI:** 10.2188/jea.JE20230023

**Published:** 2024-05-05

**Authors:** Yuya Fujii, Naruki Kitano, Yuko Kai, Takashi Jindo, Takashi Arao

**Affiliations:** 1 Physical Fitness Research Institute, Meiji Yasuda Life Foundation of Health and Welfare, Tokyo, Japan; 2 Faculty of Health and Sport Sciences, University of Tsukuba, Ibaraki, Japan

**Keywords:** physical inactivity, sitting time, office worker, social distancing

## Abstract

**Background:**

The novel coronavirus disease 2019 (COVID-19) pandemic may have reduced opportunities for engaging in physical activity (PA) and increased sedentary behavior (SB) among workers. However, most previous studies used self-reported assessments. This study aimed to examine the changes in accelerometer-measured PA and SB from before to after COVID-19 outbreak among Japanese workers.

**Methods:**

This 1-year longitudinal study used data from the annual health check-ups of workers who lived in the Tokyo metropolitan area. Baseline and follow-up data were collected from June to November of 2019 and June to November of 2020, respectively. Participants were asked to wear the accelerometer on their hip during awake hours for at least 10 days in both the surveys. Before the analysis, considering the difference in wearing time, time spent in PA and SB were converted to the percentage of wearing time. To investigate the changes in PA or SB from before to during COVID-19 outbreak, paired *t*-tests were performed.

**Results:**

Of the 757 eligible participants, 536 were included in the analysis (70.8%). Participants’ mean age was 53.3 years, 69.6% were female, and most were full-time employees. Time spent in PA decreased, regardless of weekdays or weekends, although changes on the weekends were not significant. Conversely, time spent in SB increased on both weekdays and weekends in 2020. These changes corresponded to approximately 10 minutes per day decrease in PA and increase in SB.

**Conclusion:**

Objectively measured PA decreased and SB increased by approximately 10 minutes per day after the COVID-19 outbreak among Japanese workers.

## INTRODUCTION

The novel coronavirus disease 2019 (COVID-19) initially emerged in December 2019 and was declared a global pandemic in March 2020 by the World Health Organization.^1^ In Japan, COVID-19 gradually spread since January 2020, and the National Government declared the first state of emergency on April 7, 2020.^2^ Thereafter, emergency declarations were repeatedly made from 2020 to 2022. In these declarations, social distancing measures were adopted to promote voluntary infection prevention that relied on people’s self-restraint and self-defensive consciousness, unlike the lockdown restrictions in other countries.^3^

In the working population, workstyle was greatly affected by the COVID-19 pandemic. Remote working from home became extensive.^4^^,^^5^ This change in working style had a great impact on commuting and occupational physical activity (PA).^6^^,^^7^ Rathonyi et al reviewed 39 papers related to changes in worker’s PA and sedentary behavior (SB) during the pandemic. They reported that 76.4% and 75.0% of the studies described an overall decrease and increase in the amount of PA and SB, respectively.^8^ However, most previous studies that examined physical inactivity related to the COVID-19 outbreak in workers had two major methodological limitations.^8^ First, most studies assessed PA via questionnaires, which lead to a serious problem of recall bias.^9^ In general, objective methods of PA assessment are not affected by this recall bias. Considering that personal negative feelings, which worsened during the COVID-19 pandemic,^10^ extraordinarily affected recall bias,^11^ objective methods should be used to assess PA and SB before and after the pandemic. Additionally, objective measurements, such as triaxial accelerometers, can also provide evidence of when and to what extent PA changed. Given that workers’ PA patterns may differ between weekdays and weekends, this evidence would be useful for interventions. Second, a recent systematic review indicated that nearly three-quarters of previous studies employed a cross-sectional design.^8^ Longitudinal studies should be conducted to detect intra-personal change. Thus, both restrictions should be overcome to disclose less biased changes in PA and SB among employees prior to and following the pandemic.

In addition, evidence on the changes in PA and SB after the restrictions were lifted is still lacking. Most previous studies on populations, which included the working population, examined the immediate changes under the strictest behavior restriction measures (eg, lockdown)^8^^,^^12^ owing to social urgency and time constraints. A recent longitudinal study detected changes in children’s PA after the lockdown was lifted and reported that activity levels did not recover to the pre-pandemic levels.^13^ Even in the working population, identifying the changes in PA and SB after the strictest behavior restriction measures were lifted was necessary since persistent physical inactivity over time could increase the risk of non-communicable diseases.^14^

Therefore, this study aimed to examine 1-year changes in accelerometer-measured PA and SB from before to after the COVID-19 outbreak among Japanese workers.

## METHODS

### Participants and data collection

This was a 1-year longitudinal study from the Meiji Yasuda LifeStyle (MYLS) study, a prospective cohort study that used annual health check-ups data from the Meiji Yasuda Shinjuku Medical Centre in Shinjuku, Tokyo, Japan.^15^ The inclusion criteria were those who: 1) took the optional exam using an accelerometer; 2) belonged to the life insurance company or its affiliate (clerical work), in which the researcher could identify participant’s work regulations; and 3) provided informed consent to participate in this study. Baseline data were collected from June to November 2019 before the COVID-19 outbreak. Follow-up data were obtained between June 2020, immediately after the first emergency declaration was lifted,^16^ and November 2020, immediately before the second emergency declaration was issued.^17^ There were 1,059 participants in the baseline survey, and 757 participated in the follow-up survey (follow-up rate: 71.5%). Working hours and days in the companies were from 9:00 to 17:00 from Monday to Friday; a flextime system had not been introduced. Among the participants, nobody used the remote-working system during the baseline survey, and approximately 20% of employees were introduced to work-from-home during the follow-up period (information provided by the surveyed company’s human resources department). Participants in this study were white-collar workers who received financial support from the health insurance association for their annual health check-ups. The Ethical Committee of Meiji Yasuda Life Foundation of Health and Welfare approved all the study protocols (approval no. 28006).

### Physical activity and sedentary behavior

Participants’ daily PA and SB were measured using a tri-axial accelerometer (Active style Pro HJA750-C; Omron Healthcare, Kyoto, Japan), which has been validated for PA and SB measurements.^18^^,^^19^ The accelerometer was mailed to each participant with the activity measurement instructions and a health check-up kit at least 2 weeks before the health check-up day. Participants were asked to wear the accelerometer on their hip during awake hours for at least 10 days, except during conditions that could potentially damage the device (eg, water-based activities or contact sports). The epoch length was set at 60 s and estimated metabolic equivalents of task (METs) were calculated by the developer-provided software.^19^ Non-wear time was defined as an interval of 60 consecutive minutes with activity counts below the detection limit, and a valid day was considered if data exceeded 10 hours of wear time.^20^ Participants with data for ≥2 valid weekdays and ≥1 valid weekends were analyzed.^21^ Each 60 s epoch was classified as light-intensity PA (LPA; 1.6–2.9 METs), moderate- to vigorous-intensity PA (MVPA; ≥3.0 METs), or SB (≤1.5 METs). As secondary outcomes, we also evaluated bouted MVPA (≥10 consecutive minutes with allowance for interruptions of up to 2 minutes below the MVPA threshold),^22^ prolonged SB (SB accrued in bouts ≥30 minutes),^23^^,^^24^ and step counts. Time spent engaging in each of these behaviors and step counts were aggregated per day and averaged over all the valid days. To capture the diurnal variation in activity level, hourly total PA in METs-h was calculated.

### Demographic data

To identify participants’ characteristics, their demographic information was obtained. These variables included age (continuous), sex, height, and weight (continuous), body mass index (BMI) (continuous), marital status (married or unmarried), year of education (continuous), self-rated economic status (very good, good, poor, or very poor), job type (manager, clerk, specialist, sales/service, or others), and hiring status (full-time employee or others). Office workers were those whose responses to job types as a manager, clerk, specialist, or others. BMI was calculated using measured height and weight, and the remaining variables were assessed via a self-administrated questionnaire.

### Statistical analysis

Before the analysis, considering the difference in wearing time, time spent in PA and SB were converted to the percentage of wearing time. To describe the distribution of PA and SB in 2019 and 2020, we illustrated box plots and violin plots. To investigate the changes in PA or SB from before to during COVID-19 outbreak, we performed paired *t*-tests and calculated the effect size (Cohen’s d). Analyses were conducted both overall and separately for weekdays and weekends. Diurnal pattern of total PA was illustrated using line plot and heat map. For a sensitivity analysis, all analyses were also conducted separately by sex, age (under or over 50 years old), job types (office workers or sales/service workers), and self-rated economic status (very poor/poor or good/very good). The age cutoff was set at 50 years, close to the mean age of this population (53.3 years old). All statistical analyses and visualization were performed using R version 4.0.2 (R Foundation for Statistical Computing, Vienna, Austria). Significance level was set at *P* < 0.05.

## RESULTS

There were 1,059 participants in the baseline survey and 757 in the follow-up survey (follow-up rate: 71.5%). Out of 757 eligible participants, 221 workers (29.2%) were excluded due to missing data on an accelerometer, and 536 (70.8%) were included in the analysis. Baseline characteristics are shown in eTable 1 for participants included in the analysis, as well as those not included (lost to follow-up or missing data on an accelerometer). Baseline and follow-up surveys were conducted at the same season for most participants (eFigure 1), and the average follow-up period was 378.6 (standard deviation, 36.2) days (Table 1). Participants’ mean age was 53.3 years; 69.6% were female workers; mean education year was 14.5 years (comparable to college-educated); and most were full-time employees. Participants’ characteristics of PA and SB are shown in eTable 2.

**Table 1.** Participants’ characteristics at baseline

Variables	Overall (*n* = 536)	Men (*n* = 163)	Women (*n* = 373)
Age, years, mean (SD)	53.3 (8.9)	51.9 (6.9)	53.9 (9.6)
Sex			
Male	163 (30.4%)	—	—
Female	373 (69.6%)	—	—
Height, cm, mean (SD)	161.5 (9.0)	171.9 (5.8)	157.0 (5.9)
Weight, kg, mean (SD)	59.6 (12.4)	68.5 (11.6)	55.7 (10.7)
Body mass index, kg/m^2^, mean (SD)	22.7 (3.8)	23.1 (3.4)	22.5 (4.0)
Marital status			
Married	352 (65.9%)	141 (86.5%)	211 (56.9%)
Unmarried	182 (34.1%)	22 (13.5%)	160 (43.1%)
Missing	2	0	2
Education, years, mean (SD)	14.5 (1.8)	15.9 (0.8)	13.9 (1.8)
Missing	15	3	12
Self-rated economic status			
Very good	31 (5.8%)	12 (7.4%)	19 (5.2%)
Good	364 (68.5%)	122 (74.8%)	242 (65.8%)
Poor	113 (21.3%)	24 (14.7%)	89 (24.2%)
Very poor	23 (4.3%)	5 (3.1%)	18 (4.9%)
Missing	5	0	5
Job type			
Office workers	390 (72.8%)	150 (92.0%)	240 (64.3%)
Sales/service workers	146 (27.2%)	13 (8.0%)	133 (35.7%)
Hiring status			
Full-time employee	424 (80.5%)	148 (91.9%)	276 (75.4%)
Others	103 (19.5%)	13 (8.1%)	90 (24.6%)
Missing	9	2	7
Follow-up period, days, mean (SD)	378.6 (36.2)	377.0 (34.3)	379.3 (37.1)

SD, standard deviation.

**Figure 1.**
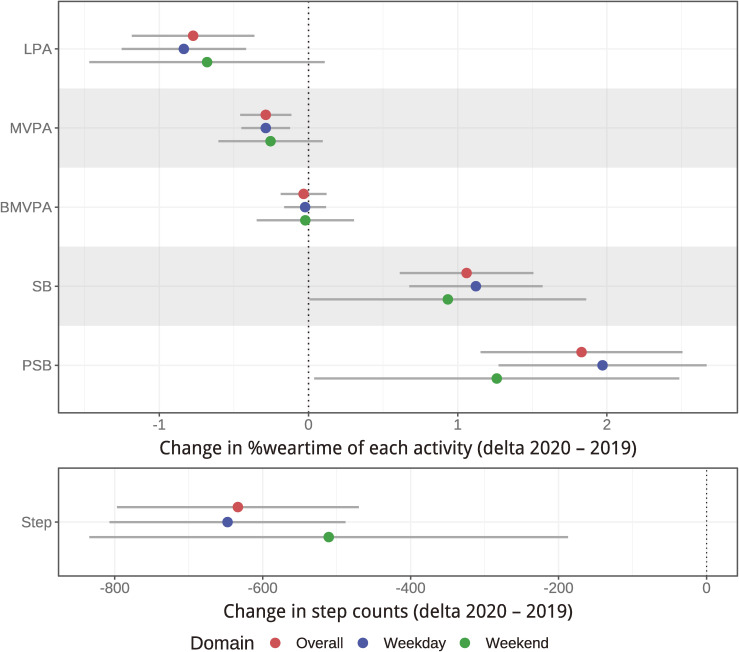
Changes in physical activity and sedentary behavior from 2019 to 2020. Points indicate the mean values and error bars indicate 95% confidence intervals. Percent wear time indicated change in the proportion of time spent in each activity relative to the time spent wearing the accelerometer. BMVPA, bouted moderate- to vigorous-intensity physical activity (ie, moderate- to vigorous-intensity physical activity lasting ≥10 min); LPA, light-intensity physical activity; MVPA, moderate- to vigorous-intensity physical activity; PSB, prolonged sedentary behavior (ie, sedentary behavior lasting ≥30 min); SB, sedentary behavior.

Figure 1 shows the changes in PA, SB, and steps from 2019 to 2020. Each point indicates the mean change of % wearing time in PA and SB. If the point was located to the left of zero, it indicated a decrease in that activity in 2020. Time spent in LPA and MVPA decreased, regardless of weekday or weekend, although changes on weekends were not significant. Conversely, time spent in SB increased on both weekdays and weekends. Specifically, when integrating LPA and MVPA, the decrease in PA corresponded to approximately 1 percentage point within the proportion of accelerometer wearing time regardless of weekdays or weekends, while SB increased by the same amount. Given that the average time of wearing on weekdays at baseline was 951 minutes, the change of 1 percentage point was taken as 9.5 minutes per day (approximately 10 minutes). No clear changes were observed in time spent in bouted MVPA on weekdays and weekends. Prolonged SB increased on both weekdays and weekends. Step counts decreased on both weekdays and weekends (200–800 steps/day). All the changes are described in eTable 3 and eFigure 2. Results from sensitivity analyses indicated that these changes in PA and SB were similar in their direction and magnitude when examined by sex, age, job types and self-rated economic status (eFigure 3, eFigure 4, eFigure 5, and eFigure 6).

**Figure 2.**
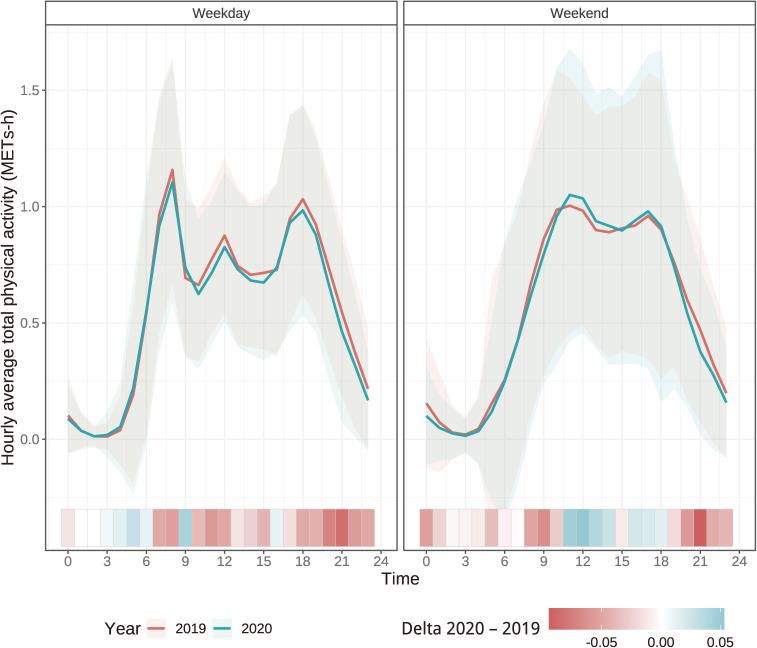
Comparison of the change in diurnal variations of total physical activity between 2019 and 2020. Shadows with the line plots indicate the range of the mean ± 1 standard deviation. In the heat map, the darker red and blue indicate a greater decrease or increase in total physical activity from 2019 to 2020, respectively.

Figure 2 shows the change in diurnal variations of total PA in 2019 (red line) and 2020 (blue line), which are plotted on weekdays and weekends separately. Shadows with the line plots indicated the range of mean ± 1 standard deviation. In the heat map, the darker red and blue indicated a greater decrease or increase in total PA from 2019 to 2020, respectively. Results on weekday showed that the heatmap from 7:00 to 24:00 was painted in red line plots, which indicated a decreasing total PA in 2020 compared to 2019. In contrast, on weekends, total PA increased from 11:00 to 18:00 and decreased after 19:00.

## DISCUSSION

This was the first study to clarify 1-year changes in accelerometer-measured PA and SB from before to after the COVID-19 outbreak in workers with fixed time. The results suggested decreased PA and increased SB from before to after the COVID-19 outbreak, mainly on weekdays. Specifically, when integrating LPA and MVPA, the decrease in PA corresponded to approximately 1 percentage point within the proportion of accelerometer wearing time regardless of weekdays or weekends, while SB increased by the same amount. Since the average wearing time on weekdays at baseline was 951 minutes, a change of 1 percentage point was taken as 9.5 minutes per day (approximately 10 minutes). Sensitivity analysis also suggested that these trends did not differ considerably by sex, age, job type, and self-rated economic status.

A previous review reported that most studies on workers suggested that PA and SB were negatively affected by the lockdown for the prevention of COVID-19.^8^ However, many studies evaluated PA and SB using questionnaires, and the issue of measurement bias was reported. In a systematic review on the impact of COVID-19 on PA in all generations, Wunsch et al emphasized the importance of utilizing objective assessments of behavior and longitudinal data for investigating the changes in PA during the pandemic.^12^ We overcame these methodological limitations using objective longitudinal data to extend prior findings of lower PA and increased SB during the pandemic.

Although we observed decreased PA and increased SB after the COVID-19 outbreak, the changes in these behaviors showed a slight difference between weekdays and weekends. On weekdays, every behavior, except for bouted MVPA, showed changes; however, on weekends, changes were found only in SB, prolonged SB, and step counts. Limited studies have examined the changes in behaviors among workers in response to COVID-19 on weekdays and weekends separately.^25^ Brusaca et al found that workers spent less time in moderate-to-vigorous intensity PA on workdays during the COVID-19 pandemic compared to before the pandemic, although behaviors during the weekends changed sparsely from pre- to during-pandemic times.^25^ Our results were partly consistent with their results. The reason why most intensity of PA did not show changes on weekends seems to be due to a large variance in these behaviors compared to weekdays (eTable 2). Even though the objective measurement can assess less biased PA on the day they are worn, much data is needed to estimate the average amount of PA on weekends, when behavior varies greatly from day to day. In our data, the average number of wearing days on weekends was only 4.5 days, which may not have been sufficient to evaluate the “habitual” behaviors on weekends. Further studies are warranted to determine the changes in PA and SB on weekends using a longer measurements period.

Regarding the diurnal changes, average total PA decreased almost the entire day after 7:00 on weekdays in 2020 compared to 2019. This decrease reflected the change in workstyle in response to pandemic restriction measures. Under the emergency measures in the Tokyo metropolitan area, employers were asked to reduce employees coming to workplace to 70% compared to before the pandemic by the National Government.^26^ Even after the state of emergency was lifted, the basic measures to prevent the transmission of the infection were continued to be thoroughly implemented. According to the surveyed company’s human resources department, only approximately 20% of the employees introduced work-from-home during the follow-up period. The remaining were asked to avoid meeting directly with working staff and business clients. Therefore, all these workstyles led to decreased total PA and increased SB during the daytime in this study. In contrast, decreased total PA after working hour (17:00) could have been due to the restrictions on using leisure facilities and holding events as pandemic prevention measures. Particularly in the Tokyo metropolitan area, a noticeable decrease in people visiting “nightlife place” has been reported.^27^ Taken together, our results suggested that Japanese workers who lived in the metropolitan area could have experienced a decline in PA before, at, and after work on weekdays. In contrast, looking at the diurnal changes in weekends, PA tended to increase during the daytime, though decreased in the evening and later. The increase in daytime activity is thought to have occurred as a “compensatory activity” for the night place restriction (eg, people started going shopping during the daytime because stores are closed at night). However, PA on weekends tended to decrease overall, suggesting that nighttime activity did not completely replace daytime activity.

In our study, the decrease/increase in PA/SB were confirmed to be approximately 10 minutes per day. This change was relatively smaller than that confirmed in the previous studies,^12^ which reported a decrease and increase in PA and SB by 23^28^ and 75 min per day^29^ after COVID-19 outbreak, respectively. There were two possible reasons behind this difference. The first reason may be the difference in methods of evaluating PA. Although we used an accelerometer to measure PA and SB, most previous studies used self-reported questionnaires, which may result in a recall bias.^9^ Questionnaires have been reported to estimate pessimistically under negative circumstances.^11^ In psychologically negative situations, such as during COVID-19 pandemic,^10^ self-reported PA may be pessimistically estimated (more in the past, less in the present). Thus, the effect size of changes in PA and SB may have been overestimated in the previous studies. The second reason was the difference of the restriction measures implemented. Most previous studies conducted surveys in the area where lockdowns were implemented.^8^^,^^12^ We conducted the survey in Tokyo where mild restrictions were places, unlike hard restrictions, such as lockdowns. Additionally, it was conducted after these restrictions had been lifted. Future studies should be conducted to compare the impact on PA and SB for each severity of restriction.

Though this study showed relatively small changes in PA and SB, it did not indicate a small impact on public health. In Japan, the Ministry of Health, Labour and Welfare established PA guidelines in 2013 and emphasized the “+10” message to add 10 minutes of PA per day.^30^ The “+10” recommendation was based on the evidence that increasing MVPA by 10 minutes a day could reduce the risk of non-communicable diseases, dementia, joint-musculoskeletal impairment, and even death by 2–8%.^31^ Given that total PA “decreased” by 10 minutes per day in our study, the population moved in the opposite direction of the “+10” recommendation (toward increased risk of disease) during the pandemic. Increasing PA at the population level by 10 minutes per day is challenging.^32^ Thus, we require further proactive interventions to increase PA for public health.

The strength of this study was that our results were suggested as essential conditions for investigating changes in behaviors during the pandemic. Therefore, these results have advantages to avoid measurement bias and provide detailed changes in PA or SB after COVID-19 outbreak in Japanese workers. However, our study has several limitations. First, external validity: this study was conducted on employees of the companies in the Tokyo metropolitan area. The majority of the participants were full-time employees with fixed working time and relatively affluent. Hence, it was unclear whether the results were applicable to people in other regions, countries, occupations, work schedule (eg, flextime), or of a different socioeconomic status. Second, although this study used an objective assessment of PA, misclassification may have occurred regarding activity intensity. In particular, the waist-mounted accelerometer could not detect a posture, which could have led to a misclassification of SB and LPA.^33^ Finally, no control group was set, as all participants had been exposed to COVID-19 outbreak, and we could not dispel the criticism that the results simply reflected a time change.

In conclusion, after the COVID-19 outbreak, PA decreased and SB increased in Japanese workers with fixed time, and the amount of change corresponded to 10 minutes per day. Furthermore, the changes in these behaviors were clearer on weekdays than on weekends and were confirmed before, at, and after working hours on weekdays. These findings could be useful information for the development of measures to prevent adverse effects of pandemic restrictions on PA and SB among workers. In particular, the country, companies, and individual workers should develop a plan to compensate for the reduction in weekday commuting, work-related PA during the day, and PA in leisure time after work, as suggested by the results of this study. Future research is required to explore ways to recover PA and SB among workers during or after the COVID-19 pandemic.

## SUPPLEMENTARY MATERIAL

eTable 1. Comparison of baseline characteristics of participants included in the analysis and lost to follow-up

eTable 2. Characteristics of physical activity and sedentary behavior among the study participants

eTable 3. Changes in physical activity and sedentary behavior for all participants

eFigure 1. Interval between baseline survey and follow-up survey for each participant. The time of the baseline survey is indicated by a red circle, and the time of the follow-up survey is indicated by a blue circle. The same month as the baseline survey is highlighted in blue (means a 1-year follow-up period).

eFigure 2. Distribution of physical activity and sedentary behavior in 2019 and 2020. Points in the boxplots indicate the mean values. IQR, interquartile range; LPA, light-intensity physical activity; Max: maximum; Min, minimum; MVPA, moderate- to vigorous-intensity physical activity; SB, sedentary behavior.

eFigure 3. Changes in physical activity and sedentary behavior from 2019 to 2020 by sex. Points indicate the mean values and error bars indicate 95% confidence intervals. Percent wear time indicated change in the proportion of time spent in each activity relative to the time spent wearing the accelerometer. BMVPA, bouted moderate- to vigorous-intensity physical activity (ie, moderate- to vigorous-intensity physical activity lasting ≥10 min); LPA, light-intensity physical activity; MVPA, moderate- to vigorous-intensity physical activity, PSB, prolonged sedentary behavior (ie, sedentary behavior lasing ≥30 min); SB, sedentary behavior.

eFigure 4. Changes in physical activity and sedentary behavior from 2019 to 2020 by age. Points indicate the mean values and error bars indicate 95% confidence intervals. Percent wear time indicated change in the proportion of time spent in each activity relative to the time spent wearing the accelerometer. BMVPA, bouted moderate- to vigorous-intensity physical activity (ie, moderate- to vigorous-intensity physical activity lasting ≥10 min); LPA, light-intensity physical activity; MVPA, moderate- to vigorous-intensity physical activity; PSB, prolonged sedentary behavior (ie, sedentary behavior lasing ≥30 min); SB, sedentary behavior.

eFigure 5. Changes in physical activity and sedentary behavior from 2019 to 2020 by job type. Points indicate the mean values and error bars indicate 95% confidence intervals. Percent wear time indicated change in the proportion of time spent in each activity relative to the time spent wearing the accelerometer. BMVPA, bouted moderate- to vigorous-intensity physical activity (ie, moderate- to vigorous-intensity physical activity lasting ≥10 min); LPA, light-intensity physical activity; MVPA, moderate- to vigorous-intensity physical activity; PSB, prolonged sedentary behavior (ie, sedentary behavior lasing ≥30 min); SB, sedentary behavior.

eFigure 6. Changes in physical activity and sedentary behavior from 2019 to 2020 by self-rated economic status. Points indicate the mean values and error bars indicate 95% confidence intervals. Percent wear time indicated change in the proportion of time spent in each activity relative to the time spent wearing the accelerometer. BMVPA, bouted moderate- to vigorous-intensity physical activity (ie, moderate- to vigorous-intensity physical activity lasting ≥10 min); LPA, light-intensity physical activity; MVPA, moderate- to vigorous-intensity physical activity; PSB, prolonged sedentary behavior (ie, sedentary behavior lasing ≥30 min); SB, sedentary behavior.
