# Supplementary material for: Changes in Accelerometer-measured Physical Activity and Sedentary Behavior From Before to After COVID-19 Outbreak in Workers
Source: J Epidemiol. 2024 May 5;34(5):247–53. doi: 10.2188/jea.JE20230023 (PMC10999519; doi:10.2188/jea.JE20230023)
Supplement: Supplementary file 1 [file je-34-247-s001.pdf]

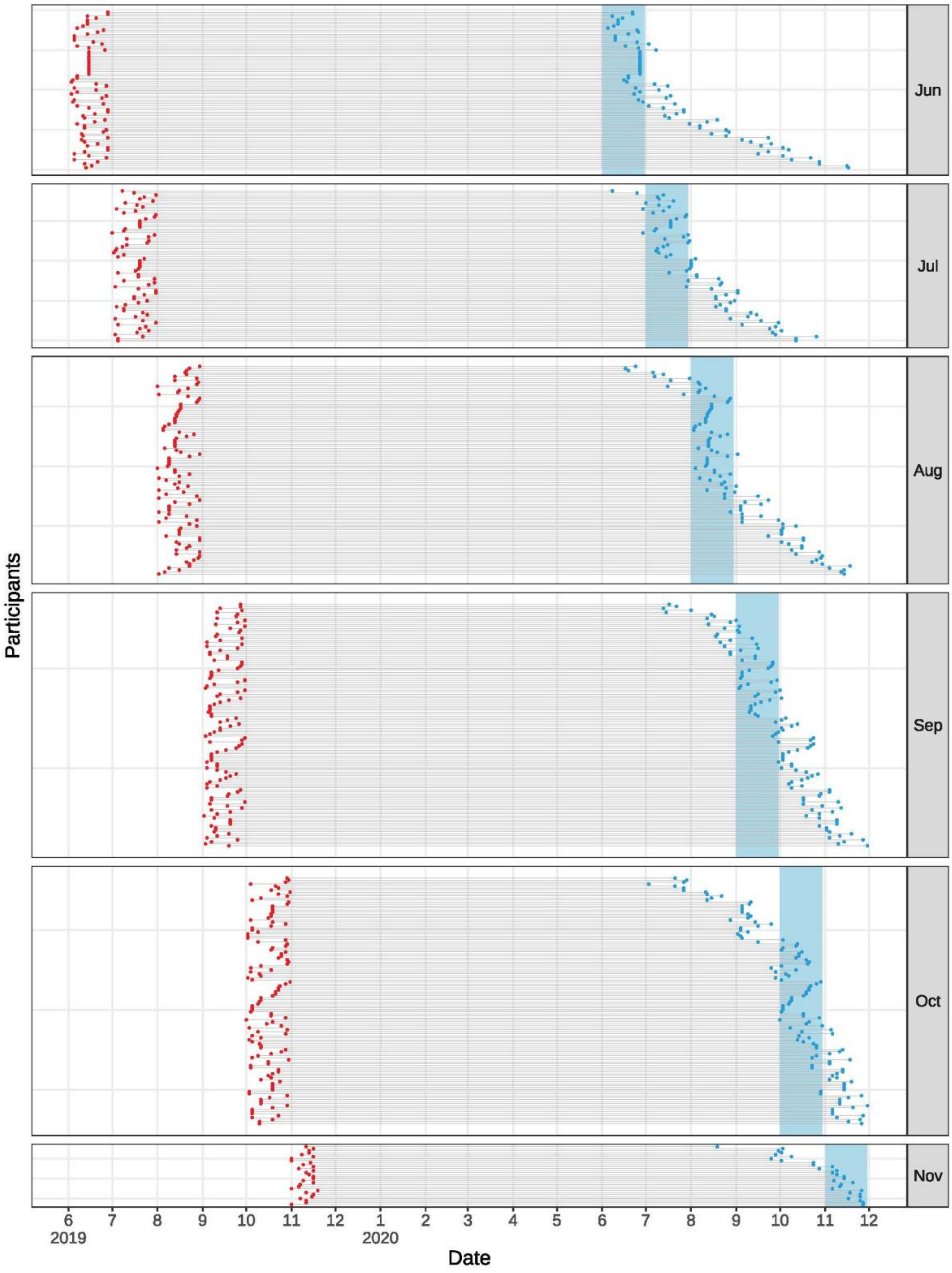

eFigure 1. Interval between baseline survey and follow-up survey for each participant. The time of the baseline survey is indicated by a red circle, and the time of the follow-up survey is indicated by a blue circle. The same month as the baseline survey is highlighted in blue (means a 1-year follow-up period).

eTable 1. Comparison of baseline characteristics of participants included in the analysis and lost to follow-up

Variables	Complete cases (n=536)	Loss-to follow-up (n =523)	*P-value* [a]
Mean (SD) age, years	53.3 (8.9)	52.0 (9.3)	0.03
Sex			0.35
Male	163 (30.4%)	174 (33.3%)	
Female	373 (69.6%)	349 (66.7%)	
Mean (SD) height, cm	59.6 (12.4)	60.8 (12.3)	0.11
Mean (SD) weight, kg	161.5 (9.0)	162.0 (8.6)	0.35
Mean (SD) body mass index, kg/m^2	22.7 (3.8)	23.0 (3.8)	0.19
Marital status			0.43
Married	352 (65.9%)	349 (68.4%)	
Unmarried	182 (34.1%)	161 (31.6%)	
Missing	2	13	
Mean (SD) year of education, year	14.5 (1.8)	14.5 (1.9)	0.85
Missing	15	39	
Self-rated economic status			0.07
Very good	31 (5.8%)	38 (7.5%)	
Good	364 (68.5%)	313 (61.5%)	
Poor	113 (21.3%)	139 (27.3%)	
Very poor	23 (4.3%)	19 (3.7%)	
Missing	5	14	
Job type			0.17
Office workers	390 (72.8%)	335 (68.6%)	
Sales/service workers	146 (27.2%)	153 (31.4%)	
Missing	0	35	
Hiring status			0.44
Full-time employee	424 (80.5%)	382 (78.3%)	
Others	103 (19.5%)	106 (21.7%)	
Missing	9	35	

SD, standard deviation.

[a] Statistical tests performed: t-test; chi-square test of independence.

eTable 2. Characteristics of physical activity and sedentary behavior among the study participants

Variables	Baseline (2019)	Follow-up (2020)
	Mean (SD)	Mean (SD)
Wearing period, days		
Weekday	12.2 (4.5)	11.8 (4.5)
Weekend	4.6 (2.5)	4.5 (2.5)
Wearing time, min/day		
Weekday	951.1 (122.7)	936.7 (131.0)
Weekend	873.6 (148.3)	868.1 (154.7)
Activity time, min/day		
Weekday		
LPA	270.5 (92.3)	259.0 (91.4)
MVPA	68.0 (25.3)	64.1 (24.4)
BMVPA[a]	27.3 (19.7)	26.6 (20.4)
SB	612.5 (126.9)	613.6 (128.5)
PSB[a]	221.0 (118.4)	236.0 (124.6)
Weekend		
LPA	276.3 (94.2)	267.8 (96.8)
MVPA	58.3 (37.3)	55.5 (36.5)
BMVPA[a]	26.4 (31.9)	25.9 (33.3)
SB	539.0 (150.5)	544.9 (161.3)
PSB[a]	257.1 (138.8)	268.5 (158.3)
Steps, step/day		
Weekday	9163.7 (2645.2)	8516.2 (2723.0)
Weekend	6965.6 (4048.9)	6454.9 (4077.3)

BMVPA, bouted moderate- to vigorous-intensity physical activity; LPA, light-intensity physical activity; MVPA, moderate- to vigorous-intensity physical activity; PSB, prolonged sedentary behavior; SB, sedentary behavior; SD, standard deviation.

[a] The total daily minutes are shown in PSB/BMVPA.

eTable 3. Changes in physical activity and sedentary behavior for all participants

		Baseline (Mean)	Follow-up (Mean)	Change[c]	95% CI			*p*-value	Cohen's d
LPA [a]	overall	29.3	28.6	-0.77	-1.18	to	-0.36	<0.01	0.09
	weekday	28.5	27.7	-0.84	-1.25	to	-0.42	<0.01	0.09
	weekend	31.8	31.2	-0.68	-1.47	to	0.11	0.09	0.06
MVPA [a]	overall	7.1	6.8	-0.29	-0.46	to	-0.12	<0.01	0.11
	weekday	7.2	6.9	-0.29	-0.45	to	-0.12	<0.01	0.11
	weekend	6.8	6.5	-0.25	-0.60	to	0.10	0.15	0.06
BMVPA[a]	overall	2.9	2.9	-0.03	-0.19	to	0.12	0.67	0.01
	weekday	2.9	2.9	-0.02	-0.16	to	0.12	0.75	0.01
	weekend	3.1	3.1	-0.02	-0.35	to	0.30	0.90	0.01
SB [a]	overall	63.6	64.6	1.06	0.61	–	1.51	<0.01	0.12
	weekday	64.2	65.4	1.12	0.68	–	1.57	<0.01	0.12
	weekend	61.4	62.3	0.93	0.01	–	1.86	0.05	0.08
PSB [a]	overall	24.5	26.3	1.83	1.15	–	2.51	<0.01	0.17
	weekday	23.0	25.0	1.97	1.27	–	2.67	<0.01	0.17
	weekend	29.0	30.3	1.26	0.04	–	2.48	0.04	0.09
Step [b]	overall	8575	7941	-634	-797	to	-470	<0.01	0.24
	weekday	9164	8516	-648	-807	to	-488	<0.01	0.24
	weekend	6966	6455	-511	-834	to	-187	<0.01	0.13

BMVPA, bouted moderate- to vigorous-intensity physical activity; CI, confidence interval; LPA, light-intensity physical activity; MVPA, moderate- to vigorous-intensity physical activity; PSB, prolonged sedentary behavior; SB, sedentary behavior.

[a] Average percentage of total wearing time.

[b] Average steps per day.

[c] The value indicates the changes in PA/SB from the baseline to follow-up.

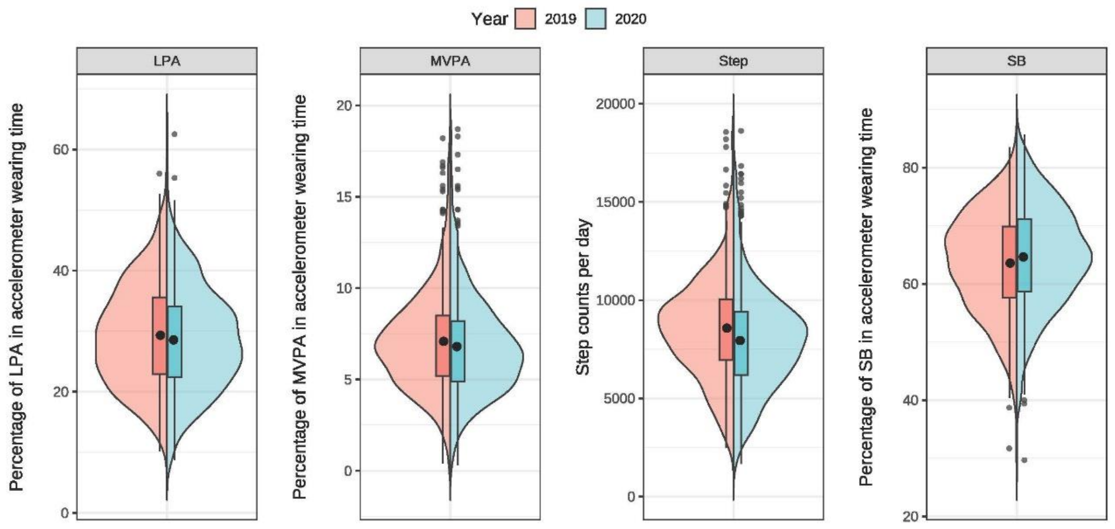

Stats	2019	2020
Max	56.0	62.5
Mean	29.3	28.6
IQR	12.6	11.7
Min	10.2	8.7

Stats	2019	2020
Max	18.2	18.7
Mean	7.1	6.8
IQR	3.3	3.3
Min	0.4	0.3

Stats	2019	2020
Max	18551.3	18618.6
Mean	8575.0	7941.5
IQR	3105.5	3216.3
Min	2484.7	1671.3

Stats	2019	2020
Max	83.5	85.7
Mean	63.6	64.6
IQR	12.3	12.5
Min	31.7	29.7

eFigure 2. Distribution of physical activity and sedentary behavior in 2019 and 2020. Points in the boxplots indicate the mean values. IQR, interquartile range; LPA, light-intensity physical activity; Max: maximum; Min, minimum; MVPA, moderate- to vigorous-intensity physical activity; SB, sedentary behavior.

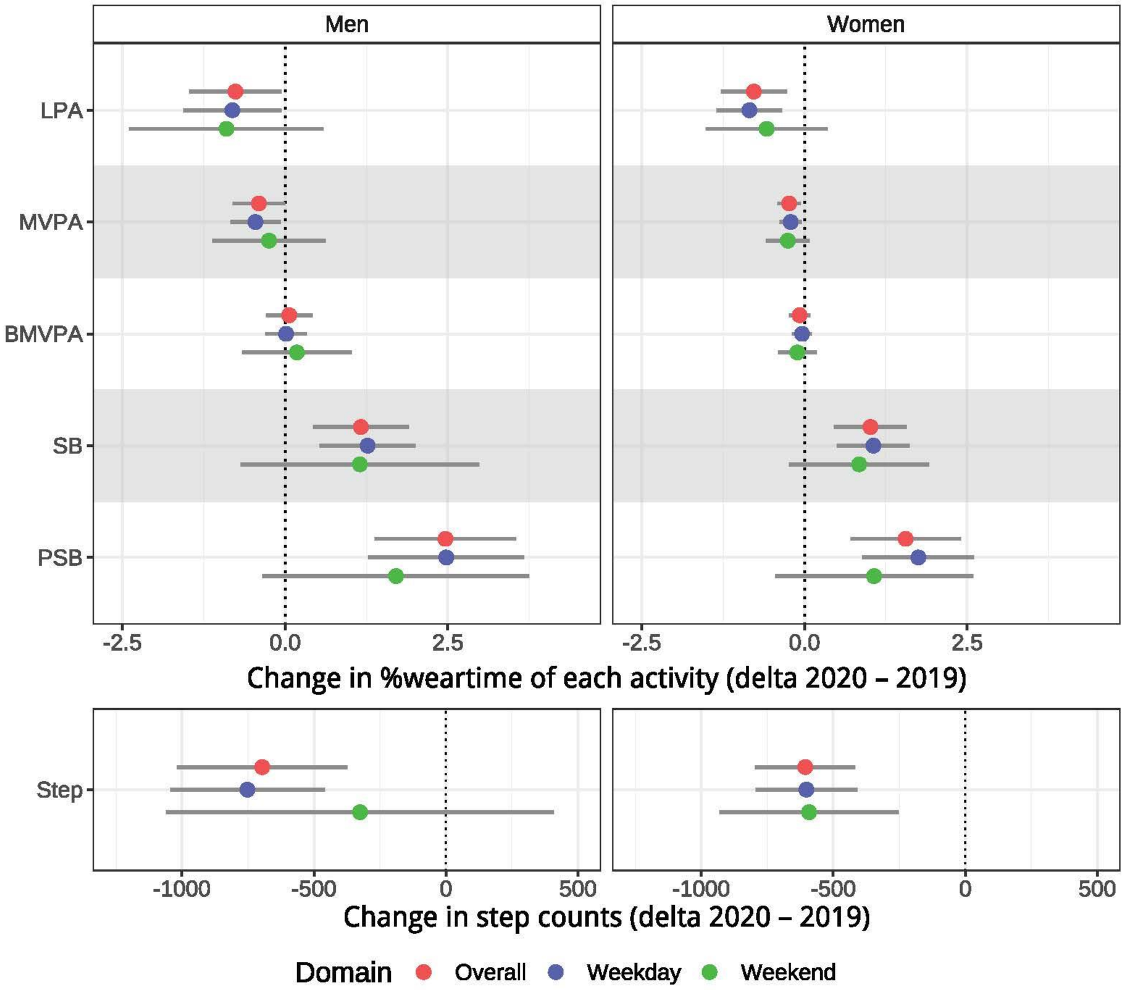

eFigure 3. Changes in physical activity and sedentary behavior from 2019 to 2020 by sex. Points indicate the mean values and error bars indicate 95% confidence intervals. Percent wear time indicated change in the proportion of time spent in each activity relative to the time spent wearing the accelerometer. BMVPA, bouted moderate- to vigorous-intensity physical activity (ie, moderate- to vigorous-intensity physical activity lasting ≥10 min); LPA, light-intensity physical activity; MVPA, moderate- to vigorous-intensity physical activity, PSB, prolonged sedentary behavior (ie, sedentary behavior lasing ≥30 min); SB, sedentary behavior.

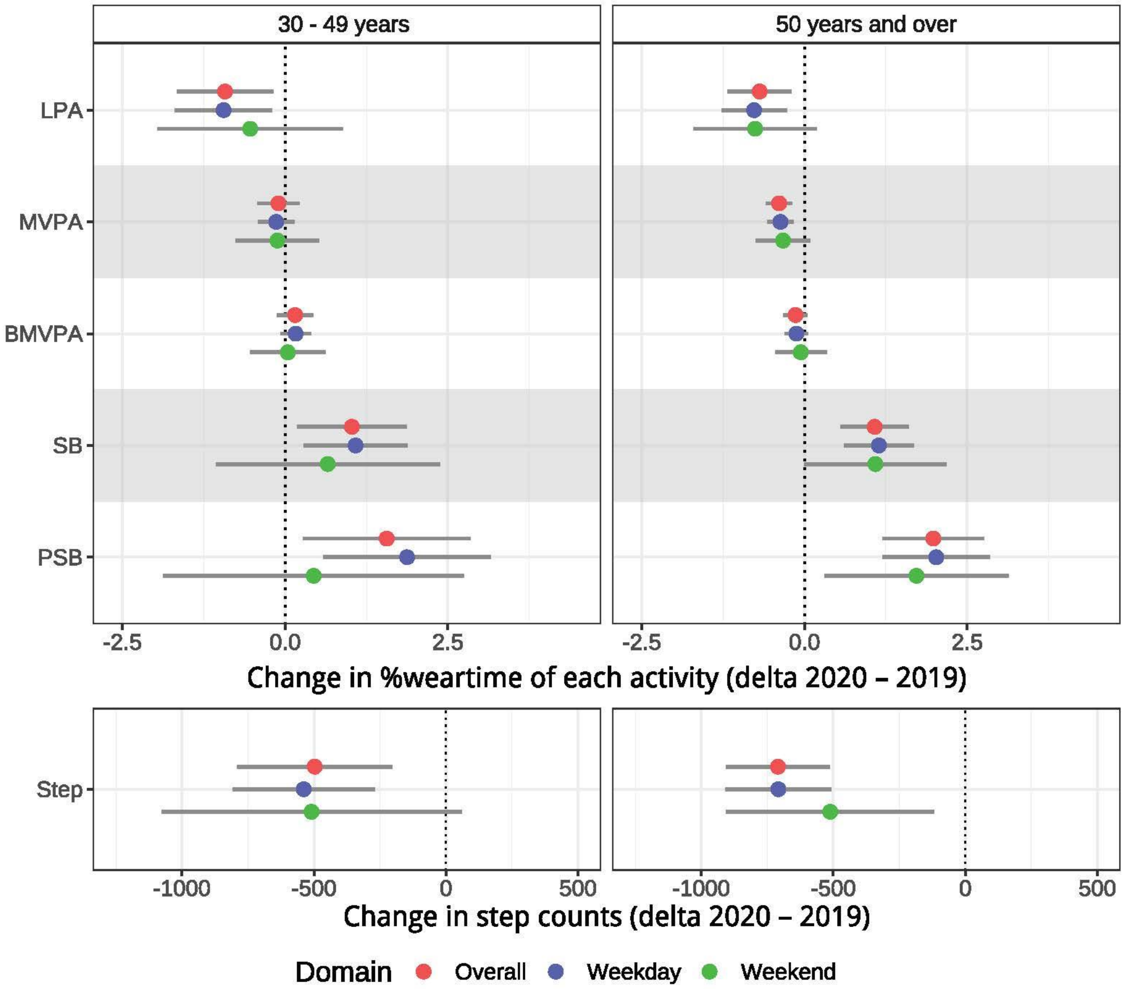

eFigure 4. Changes in physical activity and sedentary behavior from 2019 to 2020 by age. Points indicate the mean values and error bars indicate 95% confidence intervals. Percent wear time indicated change in the proportion of time spent in each activity relative to the time spent wearing the accelerometer. BMVPA, bouted moderate- to vigorous-intensity physical activity (ie, moderate- to vigorous-intensity physical activity lasting ≥10 min); LPA, light-intensity physical activity; MVPA, moderate- to vigorous-intensity physical activity; PSB, prolonged sedentary behavior (ie, sedentary behavior lasing ≥30 min); SB, sedentary behavior.

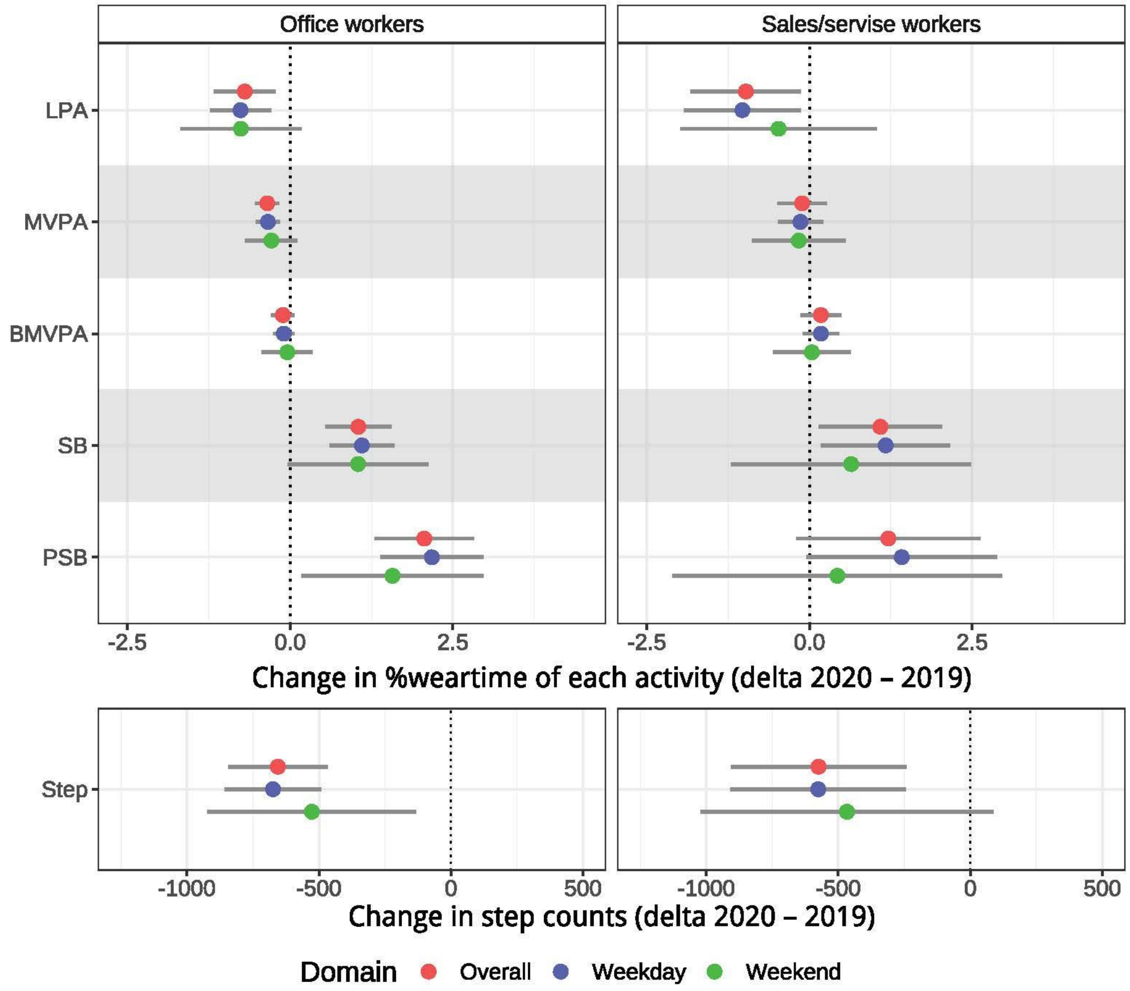

eFigure 5. Changes in physical activity and sedentary behavior from 2019 to 2020 by job type. Points indicate the mean values and error bars indicate 95% confidence intervals. Percent wear time indicated change in the proportion of time spent in each activity relative to the time spent wearing the accelerometer. BMVPA, bouted moderate- to vigorous-intensity physical activity (ie, moderate- to vigorous-intensity physical activity lasting ≥10 min); LPA, light-intensity physical activity; MVPA, moderate- to vigorous-intensity physical activity; PSB, prolonged sedentary behavior (ie, sedentary behavior lasing ≥30 min); SB, sedentary behavior.

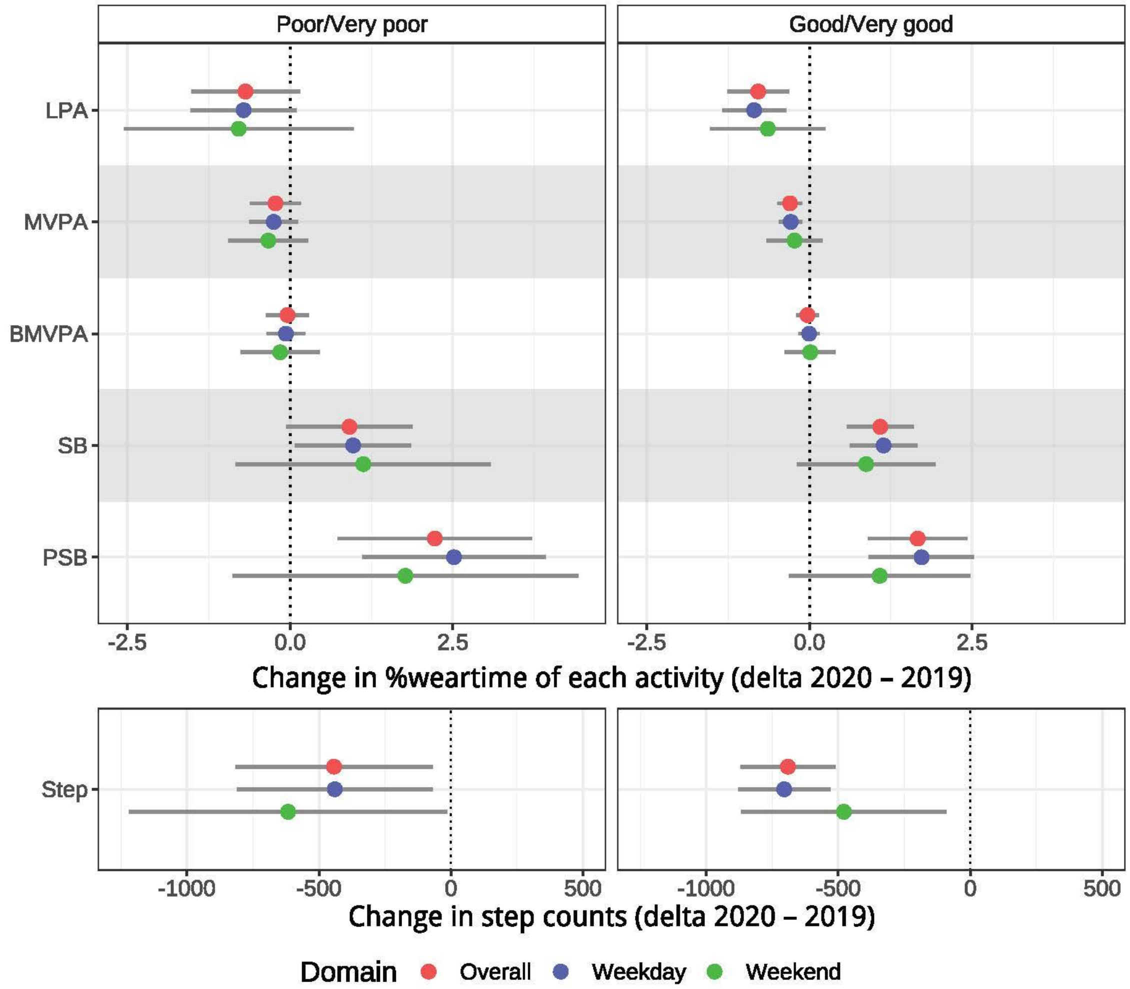

eFigure 6. Changes in physical activity and sedentary behavior from 2019 to 2020 by self-rated economic status. Points indicate the mean values and error bars indicate 95% confidence intervals. Percent wear time indicated change in the proportion of time spent in each activity relative to the time spent wearing the accelerometer. BMVPA, bouted moderate- to vigorous-intensity physical activity (ie, moderate- to vigorous-intensity physical activity lasting ≥10 min); LPA, light-intensity physical activity; MVPA, moderate- to vigorous-intensity physical activity; PSB, prolonged sedentary behavior (ie, sedentary behavior lasing ≥30 min); SB, sedentary behavior.
